# Analyzing the Responses of Enteric Bacteria to Neonatal Intensive Care Supplements

**DOI:** 10.1155/2024/3840327

**Published:** 2024-08-23

**Authors:** Megan E. Waller, Caroline J. Eichhorn, Alyssa Gutierrez, John E. Baatz, Carol L. Wagner, Katherine E. Chetta, Melinda A. Engevik

**Affiliations:** ^1^ Department of Regenerative Medicine and Cell Biology Medical University of South Carolina, Charleston, USA; ^2^ Department of Pediatrics C.P. Darby Children's Research Institute Medical University of South Carolina, Charleston, USA; ^3^ Department of Pediatrics Division of Neonatal-Perinatal Medicine Medical University of South Carolina Shawn Jenkins Children's Hospital, 10 McClennan Banks Drive, MSC 915, Charleston, SC 29425, USA; ^4^ Department of Microbiology and Immunology Medical University of South Carolina, Charleston, USA

## Abstract

In the neonatal intensive care unit, adequate nutrition requires various enteral products, including human milk and formula. Human milk is typically fortified to meet increased calorie goals, and infants commonly receive vitamin mixes, iron supplements, and less frequently, thickening agents. We examined the growth of 16 commensal microbes and 10 pathobionts found in the premature infant gut and found that formula, freshly pasteurized milk, and donated banked milk generally increased bacterial growth. Fortification of human milk significantly elevated the growth of all microbes. Supplementation with thickeners or NaCl in general did not stimulate additional growth. Vitamin mix promoted the growth of several commensals, while iron promoted growth of pathobionts. These data indicate that pathobionts in the preterm gut have significant growth advantage with preterm formula, fortified donor milk, and supplemented iron and suggest that the choice of milk and supplements may impact the infant gut microbiota.

## 1. Introduction

Approximately 10% of infants in the United States are born premature, which is defined as being born before 37 weeks of pregnancy. Preterm delivery has life-long consequences. Prematurity leads to developmental delays, vision and hearing impairment, poor somatic growth, and chronic respiratory issues [1]. In addition, prematurity is a large driver of intestinal complications, such as feeding intolerance and necrotizing enterocolitis (NEC), a severe morbidity that affects 2%–5% of all premature infants and carries a mortality rate up to 50% [2]. The annual societal economic cost, which includes medical, educational, and lost productivity associated with preterm birth, has been estimated at $25.2 billion dollars (USD). These staggering statistics highlight the urgent need for researchers and physicians to identify factors that influence perinatal outcomes.

Multiple studies have found that the preterm infant's gut microbiota is associated with health outcomes and differs dramatically from the microbiota of term infants [3–14]. In general, preterm infants have limited microbial diversity and an altered gut microbiota composition when compared to term infants [8, 13, 15]. Preterm infants have reduced levels of beneficial microbes, such as *Bifidobacterium* and *Lactobacillus* species [16, 17], and are often colonized by pathobiont microbes, such as *Enterococcus*, *Escherichia*, *Enterobacter*, and *Klebsiella* species [3–12, 14, 15]. The abundance of pathobionts, coupled with the reduction of commensal organisms, has been repeatedly linked to diseases like necrotizing enterocolitis [18–21]. For example, before the diagnosis of preterm necrotizing enterocolitis, stool samples harbor significantly more Enterobacteriaceae, particularly *Klebsiella* [20]. This has spurred researchers to search for key drivers of the preterm gut microbiota.

In addition to gestational age, diet significantly influences the infant gut microbiota composition [22, 23]. Breastfed infants are dominated by *Bifidobacterium* species [24–31]. In contrast, formula-fed infants have higher levels of Enterobacteriaceae (such as *Escherichia*, *Enterobacter*, and *Klebsiella*) and lower levels of commensal *Bifidobacterium* and *Lactobacillus* species [24, 32–34]. In the neonatal intensive care unit (NICU), breast milk is often supplemented with fortifiers, vitamins, iron, salt, and other ingredients which may alter the gut microbiota [35–37]. To date, however, few studies have examined how individual microbial strains respond to these common enteral additives.

Since establishing a healthy intestinal microbiota is key to preventing gastrointestinal disease, it is essential to understand how dietary factors and supplements influence infant gut microbes, especially pathobiont organisms that could contribute to necrotizing enterocolitis development. The goal of this study was to characterize the growth of individual gut microbes present in the infant gut in response to fresh human milk, donor breast milk, formula, and nutritional supplements commonly used in the NICU.

## 2. Methods

### 2.1. Consent and Human Milk Preparation

The Shawn Jenkin's Children's Hospital NICU obtained consent from seven mothers of infants (Medical University of South Carolina, IRB#103782). Hospital lactation specialists educated these mothers about hygienic breast pumping methods, which is a regular act performed while caring for preterm infants. The hospital's nutrition management center rigorously followed the milk-handling protocols, storing the milk expressed through these pumping methods prior to use. Expressed milk was collected fresh within 24 hours of expression and pasteurized with Holder pasteurization (HoP) for 30 minutes before immediate use in bacterial experiments to eliminate the impact of common microbes, cells, and viruses. “Fresh” was defined as milk expressed more than 2 weeks from delivery and used in experiments within 1-2 weeks of collection. Pasteurized, banked donor milk was obtained from the North Charleston Mother's Milk Bank and was frozen for several months (6–9 months) before use in experiments.

### 2.2. Milk Pasteurization and Analysis

Human milk samples were thawed slowly by placing vials at room temperature and used within 24 hours after thawing. Banked donor human milk was pasteurized at 62.5°C for 30 minutes and was not expired at the time of testing. Banked milk was stored 8–10 months before use. Fresh expressed milk samples were also pasteurized at 62.5°C before testing and pooled together in equal ratios. Milk was added in 200 *μ*L aliquots to a 96 well PCR plate, covered with a heat-sealing plastic cover, rapidly heated to 62.5°C, and then maintained at constant temperature for 30 minutes using PCR thermocycler. After heat treatments, milk was rapidly cooled to 4°C and kept on ice for immediate use for bacterial experiments. Participants' fresh milk was pooled with equal ratios for analysis. Banked donor milk, which was pasteurized prior to obtaining the sample, was used for comparison. Fresh and donor milk was analyzed using a Miris infrared milk analyzer (Uppsala, Sweden) after calibration (Table 1).

### 2.3. Bacterial Culture Conditions

*Bifidobacterium* and *Lactobacillus* species were selected based on their identification in infant feces [38–49]. The pathobionts were selected based on identification in preterm stool and NEC [38, 39, 50–53]. Bacteria growth information is found in Table 2. *Bifidobacterium* and *Lactobacillus* species were grown on de Man-Rogosa-Sharpe (MRS) agar plates while all pathobionts were grown on brain heart infusion (BHI) agar plates. Single colonies were grown in rich media (MRS for *Bifidobacterium* and *Lactobacillus* species and BHI for all other microbes) overnight at 37°C in an anaerobic chamber (Anaerobe Systems, AS-150). After overnight growth, bacteria morphology was inspected by light microscopy using 40x (Motic AE 2000) and the optical density of the cultures were taken at an optical density of 600 nm (OD_600nm_) on a Spectronic 200 Spectrophotometer (ThermoFisher). Bacteria were adjusted to an OD_600nm_ of 0.1 in chemically defined media. *Bifidobacterium* and *Lactobacillus* species were added to lactic acid bacteria-defined medium (LDM4) [54] and all the pathobionts were grown in chemically defined minimal medium (CDMM) [55, 56] (Table 2).

To test individual bacterial growth in response to fresh and donor milk, breast milk samples were collected from 4 mothers, pasteurized as described above, and then added to the bacterial cultures at a 1 : 100 dilution. The 1 : 100 dilution was based on a previous study which identified that this dilution supplied sufficient nutrients to support bacterial growth but did not affect the optical density of samples and allowed for accurate quantification of bacterial OD_600nm_ in the medium [39]. Similac® 360 formula (Abbott Nutrition, Columbus, OH), Enfamil® ferrous sulfate (Fe-In-Sol) liquid iron (15 mg Fe/mL) (Mead Johnson Nutrition Evansville, IN), Enfamil® Poly-Vi-Sol pediatric multivitamin ABCDE oral liquid (Mead Johnson Nutrition, Evansville, IN), oral NaCl solution at 4 mq/mL concentration, thickening agent (SimplyThick®, SimplyThick, LLC, St. Louis, MO), and Similac® Extensively Hydrolyzed Human Milk Fortifier (Abbott Nutrition, Columbus, OH) were provided by the Medical University of South Carolina NICU. A 1 : 100 dilution of these components was also used. Human milk fortifiers were tested at 1% concentrations in culture after addition with milk (1 : 5 vol/vol) as indicated on the package insert. Growth was recorded after anaerobic incubation for 20 hours using OD_600nm_. The experiments were each done three separate times in triplicate.

### 2.4. Statistics

The data are shown as the mean ± standard deviation (stdev). One-way analysis of variance (ANOVA) with Tukey's multiple comparison test was used to compare all groups (see statistical Tables 3 and 4). One-way ANOVA was used to determine the significance between pairwise comparisons. GraphPad was used for statistical analyses and for generating graphs (GraphPad Software, Inc., La Jolla, CA). On each graph, a “∗” indicates significance, having *p* < 0.05.

## 3. Results

**Figure 1:**
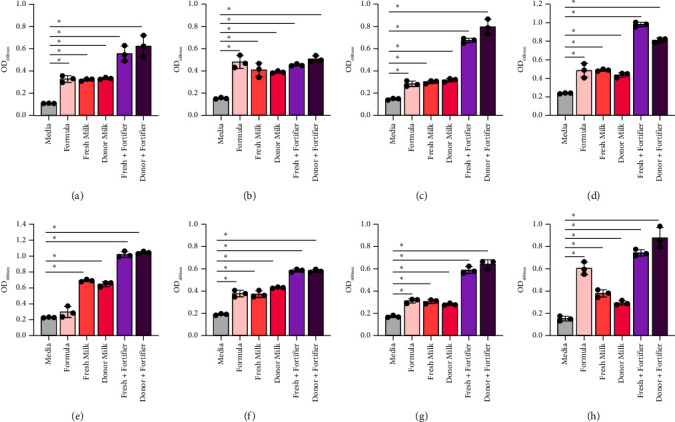
*Bifidobacterium* species: (a) *B. bifidum* ATCC 11863, (b) *B. longum* subspecies *infantis* ATCC 15697, (c) *B. longum* ATCC 55813, (d) *B. breve* ATCC 15698, (e) *B. dentium* ATCC 27678, (f) *B. animalis* Bi-07, (g) *B. gallicum* ATCC 20093, and (h) *B. angulatum* ATCC 27535 were grown in a chemically defined media, LDM4, with 1% fresh (mature) or donor milk, with or without fortification. Growth was examined at OD_600nm_ after 20 hours. Data are presented as the mean ± stdev. One-way ANOVA; ^∗^*p* < 0.05.

The microbial composition of the infant gut is influenced by the diet [57, 58]. Full-term infants that are breastfed are dominated by *Bifidobacterium* species and commonly harbor *Lactobacillus* species. Both of these microbes are considered to promote the development of the infant gut. We first examined the responses of 8 strains of *Bifidobacterium* (*B. bifidum* ATCC 11863, *B. longum* subspecies *infantis* ATCC 15697, *B. longum* ATCC 55813, *B. breve* ATCC 15698, *B. dentium* ATCC 27678, *B. animalis* Bi-07, *B. gallicum* ATCC 20093, and *B. angulatum* ATCC 27535) to formula currently used in the NICU (Figures 1(a), 1(b), 1(c), 1(d), 1(e), 1(f), 1(g), and 1(h) and Table 3). We found that 7 of the 8 *Bifidobacterium* strains had elevated growth with Similac 360 HMO-containing formula, with the exception of *B. dentium*. Compared to formula, we found that almost all *Bifidobacterium* strains had similar or improved growth with human milk (fresh milk and previously banked donor milk). The only strain that grew better with formula than human milk was *B. angulatum*. Milk is commonly fortified in the NICU to give the preterm infants extra nutrients that they need to grow. Remarkably, all *Bifidobacterium* species had improved growth in the presence of fortifier, with *B. longum* and *B. angulatum* exhibiting the most robust growth. These data indicate that all *Bifidobacterium* strains grow well with human milk, with or without fortifier. In general, we observed that human milk supported *Bifidobacterium* species more effectively, with formula outperforming human milk in only 1 of 8 strains and underperforming both types of fortified human milk in 6 of 8 strains. Overall, human milk supports the growth of the *Bifidobacterium* species better than HMO-containing formula, but there was not a significant difference between fresh milk versus frozen milk in promoting *Bifidobacterium* species growth.

**Figure 2:**
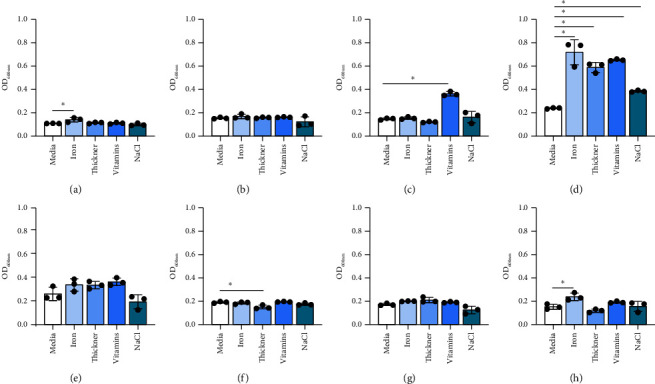
*Bifidobacterium* species: (a) *B. bifidum* ATCC 11863, (b) *B. longum* subspecies *infantis* ATCC 15697, (c) *B. longum* ATCC 55813, (d) *B. breve* ATCC 15698, (e) *B. dentium* ATCC 27678, (f) *B. animalis* Bi-07, (g) *B. gallicum* ATCC 20093, and (h) *B. angulatum* ATCC 27535 were grown in a chemically defined media, LDM4, with 1% nutritional supplements: iron, thickener, vitamin mix, and NaCl. Growth was examined at OD_600nm_ after 20 hours. Data are presented as the mean ± stdev. One-way ANOVA; ^∗^*p* < 0.05.

We also examined how other commonly provided nutrients impacted *Bifidobacterium* growth. The results were highly species dependent. Iron supplementation promoted the growth of *B. bifidum*, *B. breve*, and *B. angulatum* but did not promote the growth of the remaining five species (Figures 2(a), 2(b), 2(c), 2(d), 2(e), 2(f), 2(g), and 2(h)). The thickener increased the growth of *B. breve* but decreased the growth of *B. animalis* but did not stimulate the growth of the other six strains. Vitamin mix promoted the growth of *B. longum* and *B. breve* while NaCl elevated the growth of *B. breve* (Figures 2(a), 2(b), 2(c), 2(d), 2(e), 2(f), 2(g), and 2(h)). These findings suggest that *Bifidobacterium* exhibit species-dependent nominal growth in the presence of iron, thickener, vitamins, and NaCl.

**Figure 3:**
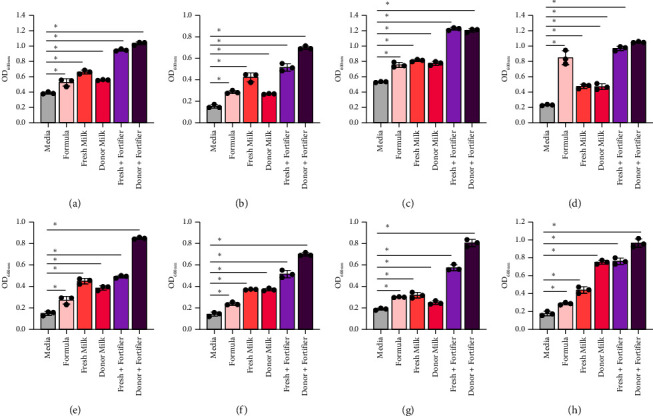
*Lactobacillus* species: (a) *L. brevis* ATCC 27303, (b) *L. acidophilus* ATCC 314, (c) *L. rhamnosus* ATCC 53163, (d) *L. plantarum* ATCC 14917, (e) *L. johnsonii* ATCC 33200, (f) *L. gasseri* ATCC 3323, (g) *L. fermentum* ATCC 14931, and (h) *L. delbrueckii* ATCC 11842 were grown in a fully defined media, LDM4, with 1% fresh (mature) or donor milk, with or without fortification. Growth was examined at OD_600nm_ after 20 hours. Data are presented as the mean ± stdev. One-way ANOVA; ^∗^*p* < 0.05.

Next, we sought to examine the responses of *Lactobacillus* species (*L. brevis* ATCC 27303, *L. gasseri* ATCC 3323, *L. acidophilus* ATCC 314, *L. plantarum* ATCC 14917, *L. johnsonii* ATCC 33200, *L. rhamnosus* ATCC, *L. fermentum* ATCC 14931, and *L. delbrueckii* ATCC 11842) to human milk and supplements (Figures 3(a), 3(b), 3(c), 3(d), 3(e), 3(f), 3(g), and 3(h) and Table 3). Similar to the *Bifidobacterium* species, all *Lactobacillus* species exhibited improved growth with HMO-containing formula and almost all species had improved growth with human milk (fresh milk and banked donor milk) compared to formula. Fortified fresh and donor milk supported robust growth of *Lactobacillus* species and this growth was higher than nonfortified milk (7 of 8 strains). These data indicate that fortified milk uniformly supports the growth of *Lactobacillus* species.

**Figure 4:**
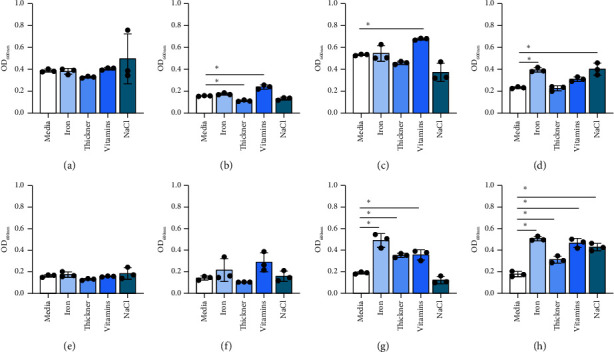
*Lactobacillus* species: (a) *L. brevis* ATCC 27303, (b) *L. acidophilus* ATCC 314, (c) *L. rhamnosus* ATCC 53163, (d) *L. plantarum* ATCC 14917, (e) *L. johnsonii* ATCC 33200, (f) *L. gasseri* ATCC 3323, (g) *L. fermentum* ATCC 14931, and (h) *L. delbrueckii* ATCC 11842 were grown in a fully defined media, LDM4, with nutritional supplements. Growth was tested at OD_600nm_ after 20 hours. Data are presented as the mean ± stdev. One-way ANOVA; ^∗^*p* < 0.05.

When growth of *Lactobacillus* was examined in response to the presence of specific nutrients, species-dependent effects were observed. Iron promoted the growth of *L. plantarum*, *L. fermentum*, and *L. delbrueckii* but did not stimulate the growth of the other five species (Figures 4(a), 4(b), 4(c), 4(d), 4(e), 4(f), 4(g), and 4(h)). Thickener decreased the growth of *L. acidophilus* but increased the growth of *L. fermentum* and *L. delbrueckii*. Vitamin mix elevated the growth of several *Lactobacillus* species, including *L. acidophilus*, *L. rhamnosus*, *L. fermentum*, and *L. delbrueckii* (Figures 4(a), 4(b), 4(c), 4(d), 4(e), 4(f), 4(g), and 4(h)). Interestingly, NaCl elevated *L. plantarum* and *L. delbrueckii* growth but did not affect the other *Lactobacillus* species. These data indicate that commensal bacteria grow well in human milk regardless of the fortification status and that several commensals respond with elevated growth to vitamins.

**Figure 5:**
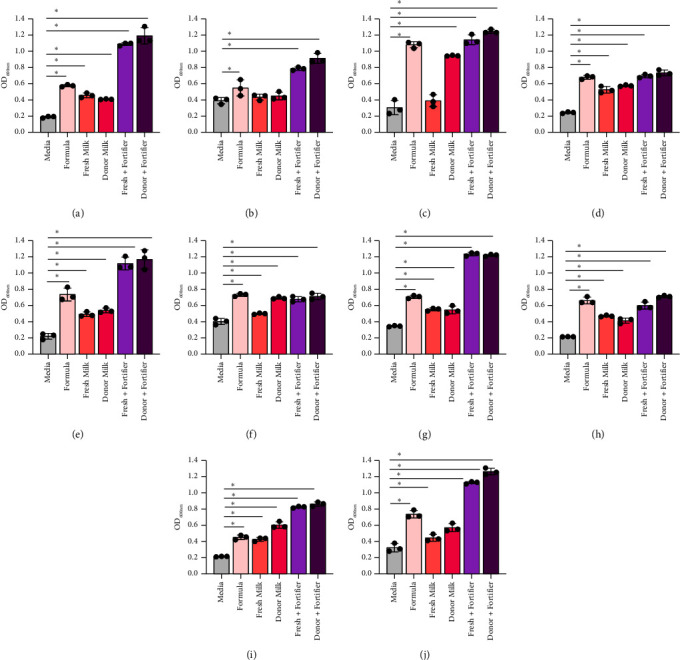
Pathobiont species in response to fresh (mature) milk. (a) *S. epidermidis* ATCC 51025, (b) *S. agalactiae* ATCC 13813, (c) *P. aeruginosa* ATCC CB1, (d) P. *vulgaris* CB1, (e) *E. cloacae* ATCC 2701, (f) *E. coli* K12, (g) *A. baumannii* ATCC 747, (h) *E. faecalis* ATCC 29212, (i) *K. pneumoniae* CB1, and (j) *K. aerogenes* NCMB 10102 were grown in a fully defined media, CDMM, with 1% fresh or donor milk, with or without fortification. Growth was examined at OD_600nm_ after 20 hours. Data are presented as the mean ± stdev. One-way ANOVA; ^∗^*p* < 0.05.

The preterm infant gut is known to harbor more pathobionts than the guts of infants born full term. Preterm infants are commonly colonized by *Klebsiella*, *Enterobacter*, *Escherichia*, *Staphylococcus*, *Streptococcus*, *Enterococcus*, *Acinetobacter*, among others. To address how pathobionts respond to milk and nutrients, 10 pathobionts that are commonly found in the preterm infant gut were examined. Specifically, growth was examined for *Staphylococcus epidermidis* ATCC 51025, *Streptococcus agalactiae* ATCC 13813, *Pseudomonas aeruginosa* CB1, *Proteus vulgaris* CB1, *Enterobacter cloacae* ATCC 2701, *Escherichia coli* K12, *Acinetobacter baumannii* ATCC 747, *Enterococcus faecalis* ATCC 29212, *Klebsiella pneumoniae* CB1, and *Klebsiella aerogenes* NCMB 10102 in response to milk, formula, and other nutrients. It was found that all pathobionts grew well in formula, with *P. aeruginosa* exhibiting the highest growth in formula (>5-fold change) (Figures 5(a), 5(b), 5(c), 5(d), 5(e), 5(f), 5(g), 5(h), 5(i), and 5(j) and Table 4). Eight of the 10 pathobionts grew in fresh human milk while 9 of the 10 pathobionts grew in donor milk. Interestingly, 7 of the 10 microbes grew better in formula than in fresh milk (Figures 5(b) and 5(c)). *P. vulgaris*, *E. cloacae*, *A. baumannii*, *E. faecalis*, *K. pneumoniae*, and *K. aerogenes* had decreased growth with fresh milk when compared to donor milk, suggesting that an antibacterial component is missing or is reduced in donor banked milk. Consistent with the commensal data, we found that all pathobionts had elevated growth when the milk was fortified.

**Figure 6:**
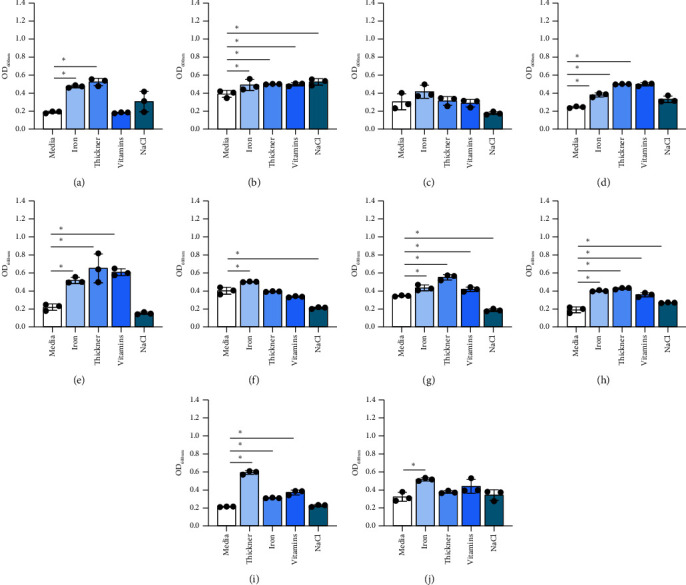
Pathobiont species in response to fresh (mature) milk. (a) *S. epidermidis* ATCC 51025, (b) *S. agalactiae* ATCC 13813, (c) *P. aeruginosa* ATCC CB1, (d) P. *vulgaris* CB1, (e) *E. cloacae* ATCC 2701, (f) *E. coli* K12, (g) *A. baumannii* ATCC 747, (h) *E. faecalis* ATCC 29212, (i) *K. pneumoniae* CB1, and (j) *K. aerogenes* NCMB 10102 were grown in a fully defined media, CDMM, with 1% nutritional supplements: iron, thickener, and vitamin mix. Growth was examined at OD_600nm_ after 20 hours. All data are presented as the mean ± stdev. One-way ANOVA; ^∗^*p* < 0.05.

Finally, the growth of pathobionts was assessed as a function of additional nutrients found in the NICU. Iron promoted the growth of all pathobionts with the exception of *P. aeruginosa* (Figures 6(a), 6(b), 6(c), 6(d), 6(e), 6(f), 6(g), 6(h), 6(i), and 6(j)). Thickener also promoted the growth of multiple pathobionts, including *S. epidermidis*, *S. agalactiae*, *P. vulgaris*, *E. cloacae*, *A. baumannii*, *E. faecalis*, and *K. pneumoniae*. The vitamin mix supported the growth of fewer species, namely, *S. agalactiae*, *P. vulgaris*, *E. cloacae*, *A. baumannii*, *E. faecalis*, and *K. pneumoniae*. NaCl only promoted the growth of *S. agalactiae* and *E. faecalis* but instead reduced the growth of *E. coli* and *A. baumannii*. These results indicate that fortified milk and iron significantly enhance the growth of pathobionts.

## 4. Discussion

The data presented herein indicate that after pasteurization, donor milk (which was previously frozen and banked) and fresh milk (stored less than one week and expressed at an early postpartum period) whether fortified or unfortified, supported the growth of commensal *Bifidobacterium* and *Lactobacillus* species. For most commensal microbes, human milk supported more growth than formula. In addition, half of the commensal microbes examined had elevated growth when cultured in a vitamin mix commonly used in the NICU. However, while these nutritional interventions support the growth of commensal microbes, they also support the growth of pathobionts. Most pathobionts exhibited a 2–4-fold increase in growth with formula. In contrast, fresh milk resulted in minimally elevated pathobiont growth, while donor milk more robustly supported pathobiont growth. Unexpectedly, fortifier significantly enhanced the ability of microbes to grow with fresh and donor milk while iron supported the growth of mostly pathobionts. These data indicate that nutritional supplements commonly administered in the NICU support the growth of both commensal and pathobiont microbes.

One of the primary conclusions of this study is that fortifier elevates the growth of pathobionts, particularly in the setting of fresh milk. This is consistent with a recent clinical trial which found that human milk-based fortifiers elevated the levels of fecal Enterobacteriaceae in very low-birth-weight preterm infants [59]. These results support the concept that fortifiers may alter the antimicrobial activity of the milk and provide trace metals that support pathobionts. A previous study has shown that enriching mothers' milk with fortifiers containing iron can lower the antimicrobial activity of milk against *E. coli*, *S. aureus*, *Enterobacter sakazakii*, and Group B *Streptococcus* [60]. Fortifiers are also enriched in trace metals, such as zinc and magnesium. Pathobionts such as *A. baumannii*, *E. coli*, *Proteus* species, and *S. agalactiae* have multiple transport pathways for zinc utilization [61]. Pathobionts also have multiple routes for magnesium uptake as magnesium supports the growth of several pathobionts [62]. Magnesium may be particularly high in preterm infants due to decreased magnesium uptake by the host transporter NRAMP1. Fatimah et al. found that babies that were not exclusively breastfed had lower levels of NRAMP1 [63]. Low levels of NRAMP1 could contribute to a larger pool of magnesium in the gut and further support the growth of pathobionts upon introduction of fortifier. These results are consistent with large cohort studies that have observed large shifts in the gut microbiome after fortifier additions to the enteral diet of preterm infants [64].

An unexpected finding was that banked donor milk supports the growth of select pathobionts (e.g., *Klebsiella*, *Pseudomonas*, and *E. coli*) more so than both groups of fresh milk even though both milk groups underwent Holder pasteurization. This may be due to molecular or structural changes resulting from the storing, freezing, and/or thawing of the milk. Freezing and thawing solutions are known to alter proteins and peptides and it is possible that compounds could aggregate when the milk is frozen and no longer be effective. Long-term storage can generate fatty-acid protein complexes through the release of free fatty acids which may alter the function of milk proteins and antimicrobial activity of milk [65, 66]. Denatured proteins could consist of lactoferrin, lysozyme, or immunoglobulins. In addition to storage, the timing of milk expression may also contribute to our observed phenotype. More studies will be required to fully tease out the mechanisms by which donor milk supports the growth of certain pathobionts.

Early milk, specifically colostrum, has more lactoferrin than mature milk as lactoferrin decreases over time [67]. Consistent with this finding, measurements performed for this study demonstrated higher total protein concentrations in fresh milk samples compared to banked donor milk. One possible explanation for this difference is that lactoferrin, an iron-binding protein, may be reduced or altered in donor milk and this may contribute to elevated growth by pathobionts, a concept that is supported by our nutrient analysis of the milk used in this study (Table 1). In the fresh milk experiments, true protein content was higher than in the donor milk in both cohorts used for experiments.

Limitations of this study include the use of a simplified design that examined single-strain response to singular supplements (with the exception that fortifiers were added directly to milk for testing) and a small sample size of infant stool. This study does not replace large cohorts that have examined microbiome changes and complex interactions between microbial communities in the preterm gut in response to diets in the NICU. In practice, enteral supplements are often given as a combination and on top of fortification in the infant diet, which may have confounding effects. Despite this limitation, we believe this study adds to our mechanistic understanding of how individual supplements may be contributing to the general microbial shifts we have observed in larger studies.

In this study, we pooled milk samples from different donors to draw broad conclusions about bacterial growth responses to human milk. Using this method, we are unable to identify if variations in human milk influences bacterial growth. For example, it is well documented that secretor status of the mother influences the infant gut microbiota composition, particularly the levels of *Bifidobacterium* species [68–71]. Other factors in human breast milk such as antimicrobial proteins, lysozyme, lipid profiles, and human milk oligosaccharides (HMOs) can also influence bacterial growth [72–75]. Future studies which include detailed information about the composition of the milk samples would be valuable to dissect bacterial responses to varying components in human milk.

Another limitation of this work is that we did not consider donor diversity. A recent study from Butts et al. analyzed milk samples from women of Asian, Māori, Pacific Island, and European ethnicity and found that there was no difference between the mothers of different ethnicities in terms of their macronutrient (protein, fat, carbohydrate, and moisture) content [76]. However, they did identify that the breast milk of Asian mothers contained significantly higher levels of polyunsaturated fatty acids (PUFAs), omega-3 (n-3) and omega-6 (n-6) fatty acids, docosahexaenoic acid (DHA), and linoleic acids and the breast milk of from mothers of Māori and Pacific Island decent had lower levels of arachidonic acid [76]. They also noted that the diet was very different between the ethnic groups and speculated that this could impact milk composition. Consistent with this work, other groups have found that diet significantly impact the profiles of breast milk. Charbonneau et al. analyzed a human cohort of Malawian women and identified that that there was decreased total, sialylated, and fucosylated HMOs in the milk of mothers with undernourished and growth-stunted infants [77]. Ellsworth et al. examined mothers in the United States and found that mothers with overweight/obese infants had higher milk insulin, dihomo-gamma-linolenic, adrenic, and palmitic acids and reduced conjugated linoleic and oleic acids compared with mothers of normal weight infants [78]. Another study by Seferovic et al. used a crossover study design with mothers in the United States to examine the impact of diet on HMO and microbiome profiles [75]. One group of women was randomized to first glucose or galactose as their sole carbohydrate source (Glu/Gal cohort), while another group of women was randomized to a high fat diet as their energy source (Carb/Fat cohort). This study found that mothers receiving a galactose or high fat diet had a significant increase in milk HMOs and unique breast milk microbial profiles [75]. These studies suggest that both ethnicity and diet may play a large role in determining the composition of mother's milk. In the future, it would be valuable to incorporate these parameters into studies examining how gut bacteria respond to milk.

In addition, this study does not account for the origins of the gut microbiota that may pre-exist before nutrients are delivered or the fact that the gut microbiota is a complex community that participates in cross-feeding and bacterial-bacterial inhibition. Along these same lines, mothers' milk has a naturally large probiotic load which is continuously delivered when a preterm infant is receiving fresh unpasteurized human milk. Microbial interactions or microbial-epithelial cross talk may be playing a larger role than nutrient-microbe interactions in shaping the microbiome. Further studies are needed to define these mechanisms.

Aside from diet, there are other factors which influence the infant gut microbiota. These factors include antibiotic use, medications, gestational age, mode of delivery, and environment [79]. Preterm infants are traditionally nursed in sanitary incubators, have limited contact with the mother's skin, commonly receive antibiotics, and have restricted breastmilk intake [80, 81]. These factors likely have a significant impact on the gut microbiota composition of preterm infants. Yang et al. recently identified that the ability of mothers to care for their preterm infants also impacts the newborn gut microbiota [81]. Yang et al. conducted a multicenter prospective cluster-randomized controlled trial on the effect of family integrated care (FICare) on premature infants in China [81, 82]. Both the control and FlCare parents received education and coaching sessions for infant care, but only FlCare mothers were allowed to enter the NICU for direct infant care for 2 weeks. The FICare trial revealed that FICare improved the richness and diversity of the premature infant gut microbiome. Interestingly, this microbiome was more similar to that in term infants in the NICU who were breastfed and did not undergo surgery. This study indicates that mother-child interaction significantly impacts the gut microbiota and could be added to nutritional strategies to improve the wellbeing of preterm infants.

In conclusion, these series of experiments demonstrate universal trends, showing that commensals and pathobionts grow rapidly in fortified human milk, more so than unfortified sources. Overall, vitamins and thickeners did not significantly affect pathobionts' or commensals' growth in a single direction but rather had strain-specific differences. Iron seemed to increase the growth of pathobionts more than commensals. This study supports previous data highlighting the antibacterial qualities of fresh and early human milk and also aligns with new studies that point to mothers' milk as a key influencer of the microbiota. While diverse communities of these microbes should be ultimately tested together, the single-strain microbial response to human milk and fortifier gives insight into how enteral supplements could potentially affect microbiome-related infant health outcomes.

## Figures and Tables

**Table 1:** Milk characteristics and macronutrient content.

	Banked donor milk *n* = 2	Mother's milk (fresh) *n*=4^∗^
Gestational age (weeks)	Term	37 ± 2
Pasteurization	Holder type	Holder type
Frozen at one point	Yes	No
Storage age and temperature^†^	6–8 months, −20°C	<24 hours, 4°C
Postpartum day	Unknown	18, 19, 28, 81
Fat (g/dL)	2.8 ± 1.0	4.7
Crude protein (g/dL)	1.1 ± 0.2	1.5
Carbohydrate (g/dL)	7.8 ± 0.4	8.2
Total solute (g/dL)	11.9 ± 1.6	14.6
Energy (kcal/dL)	62 ± 11.3	83
True protein (g/dL)	0.8 ± 0.1	1.2

^∗^Four mothers participated in this study. Fresh milk was pooled in groups of 4 donors over three independent experiments in equal volume ratios for testing. ^†^Based on expiration date of banked donor milk.

**Table 2:** Bacterial strains and growth conditions used in this study.

Bacteria	Strain	Rich media	Defined media
*B. bifidum*	ATCC 11863	MRS	LDM4
*B. longum*	ATCC 55813	MRS	LDM4
*B. infantis*	ATCC 15697	MRS	LDM4
*B. dentium*	ATCC 27678	MRS	LDM4
*B. gallicum*	ATCC 20093	MRS	LDM4
*B. angulatum*	ATCC 27535	MRS	LDM4
*B. breve*	ATCC 15698	MRS	LDM4
*B. animalis*	Bi-07	MRS	LDM4
*L. brevis*	ATCC 27303	MRS	LDM4
*L. gasseri*	ATCC 3323	MRS	LDM4
*L. acidophilus*	ATCC 314	MRS	LDM4
*L. plantarum*	ATCC 14917	MRS	LDM4
*L. johnsonii*	ATCC 33200	MRS	LDM4
*L. rhamnosus*	ATCC 53163	MRS	LDM4
*L. fermentum*	ATCC14931	MRS	LDM4
*L delbrueckii*	ATCC 11842	MRS	LDM4
*S. epidermidis*	ATCC 51025	BHI	CDMM
*S. agalactiae*	ATCC 13813	BHI	CDMM
*P. vulgaris*	CB1	BHI	CDMM
*E. coli*	K12	BHI	CDMM
*P. aeruginosa*	CB1	BHI	CDMM
*A. baumannii*	ATCC 747	BHI	CDMM
*E. cloacae*	ATCC 2701	BHI	CDMM
*E. faecalis*	ATCC 29212	BHI	CDMM
*K. aerogenes*	NCMB 10102	BHI	CDMM
*K. pneumoniae*	CB1	BHI	CDMM

**Table 3:** Statistic analysis of commensal microbes.

Bacterial strain	Media vs. Formula	Media vs. Fresh Milk	Media vs. Donor Milk	Media vs. Fresh + Fortifier	Media vs. Donor + Fortifier	Formula vs. Fresh Milk	Formula vs. Donor Milk	Formula vs. Fresh + Fortifier	Formula vs. Donor + Fortifier	Fresh Milk vs. Donor Milk	Fresh Milk vs. Fresh + Fortifier	Fresh Milk vs. Donor + Fortifier	Donor Milk vs. Fresh + Fortifier	Donor Milk vs. Donor + Fortifier	Fresh + Fortifier vs. Donor + Fortifier	Media vs. Iron	Media vs. Thickner	Media vs. Vitamins	Media vs. NaCl	Iron vs. Thickner	Iron vs. Vitamins	Iron vs. NaCl	Thickner vs. Vitamins	Thickner vs. NaCl	Vitamins vs. NaCl
*B. bifidum* ATCC 11863	0.002	0.0026	0.0017	<0.0001	<0.0001	>0.9999	>0.9999	0.0015	0.0001	0.9997	0.0012	0.0001	0.0018	0.0002	0.5826	0.0282	0.9679	0.9992	0.7884	0.0741	0.0405	0.0051	0.9943	0.448	0.6621
*B. infantis* ATCC 15697	<0.0001	<0.0001	<0.0001	<0.0001	<0.0001	0.2431	0.0931	0.9426	0.9368	0.9872	0.6851	0.0579	0.3502	0.0204	0.5022	0.9247	0.9993	0.9925	0.4004	0.9764	0.9948	0.1337	0.9997	0.3002	0.2319
*B. longum* ATCC 55813	0.002	0.0007	0.0003	<0.0001	<0.0001	0.9817	0.7692	<0.0001	<0.0001	0.9846	<0.0001	<0.0001	<0.0001	<0.0001	0.0047	0.9966	0.6616	<0.0001	0.9345	0.4726	<0.0001	0.992	<0.0001	0.2794	<0.0001
*B. breve* ATCC 15698	<0.0001	<0.0001	0.0002	<0.0001	<0.0001	>0.9999	0.598	<0.0001	<0.0001	0.5077	<0.0001	<0.0001	<0.0001	<0.0001	0.0009	<0.0001	<0.0001	<0.0001	0.0407	0.0746	0.5601	0.0001	0.5909	0.0051	0.0007
*B. dentium* ATCC 27678	0.2471	<0.0001	<0.0001	<0.0001	<0.0001	<0.0001	<0.0001	<0.0001	<0.0001	0.6412	<0.0001	<0.0001	<0.0001	<0.0001	0.9578	0.3537	0.3809	0.1421	0.461	>0.9999	0.9611	0.0275	0.9475	0.0302	0.0101
*B. animalis* BiC 2	<0.0001	<0.0001	<0.0001	<0.0001	<0.0001	0.9996	0.0422	<0.0001	<0.0001	0.0255	<0.0001	<0.0001	<0.0001	<0.0001	0.9999	0.9533	0.0037	0.982	0.3317	0.0102	0.7387	0.694	0.0018	0.076	0.1592
*B. gallicum* ATCC 20093	0.0001	0.0002	0.0014	<0.0001	<0.0001	0.9996	0.6335	<0.0001	<0.0001	0.799	<0.0001	<0.0001	<0.0001	<0.0001	0.1518	0.3749	0.1544	0.6984	0.0639	0.9629	0.9712	0.0036	0.7231	0.0015	0.0087
*B. angulatum* ATCC 27535	<0.0001	0.0013	0.0414	<0.0001	<0.0001	0.0014	<0.0001	0.0438	0.0002	0.3538	<0.0001	<0.0001	<0.0001	<0.0001	0.056	0.0219	0.5596	0.4545	>0.9999	0.0023	0.2972	0.0258	0.0506	0.5019	0.51
*L. brevis* ATCC 27303	0.0002	<0.0001	<0.0001	<0.0001	<0.0001	0.0003	0.6127	<0.0001	<0.0001	0.0028	<0.0001	<0.0001	<0.0001	<0.0001	0.0112	>0.9999	0.9515	0.9995	0.7081	0.968	0.9982	0.6638	0.886	0.3392	0.8173
*L. acidophilus* ATCC 314	0.0003	<0.0001	0.0009	<0.0001	<0.0001	0.0002	0.9551	<0.0001	<0.0001	<0.0001	0.0058	<0.0001	<0.0001	<0.0001	<0.0001	0.3927	0.0053	<0.0001	0.1193	0.0004	0.0003	0.0069	<0.0001	0.3135	<0.0001
*L. rhamnosus* ATCC 53163	<0.0001	<0.0001	<0.0001	<0.0001	<0.0001	0.0528	0.8243	<0.0001	<0.0001	0.3406	<0.0001	<0.0001	<0.0001	<0.0001	0.8417	0.9981	0.4199	0.0339	0.018	0.2902	0.0535	0.0115	0.0023	0.2748	0.0002
*L. plantarum* ATCC 14917	<0.0001	0.0002	0.0003	<0.0001	<0.0001	<0.0001	<0.0001	0.0466	0.0011	>0.9999	<0.0001	<0.0001	<0.0001	<0.0001	0.2625	0.0004	0.9985	0.0566	0.0003	0.0003	0.0377	0.997	0.0369	0.0002	0.0226
*L. johnsonii* ATCC 33200	0.0002	<0.0001	<0.0001	<0.0001	<0.0001	<0.0001	0.0005	<0.0001	<0.0001	0.0368	0.2676	<0.0001	0.0009	<0.0001	<0.0001	0.9857	0.5955	0.9991	0.8638	0.3405	0.9418	0.9886	0.7308	0.1796	0.7471
*L. gasseri* ATCC 3323	0.0004	<0.0001	<0.0001	<0.0001	<0.0001	<0.0001	<0.0001	<0.0001	<0.0001	>0.9999	<0.0001	<0.0001	<0.0001	<0.0001	<0.0001	0.6642	0.9551	0.1204	0.998	0.3129	0.6642	0.821	0.0419	0.8573	0.1864
*L. fermentum* ATCC 14931	0.0005	0.0001	0.05	<0.0001	<0.0001	0.9223	0.1103	<0.0001	<0.0001	0.0223	<0.0001	<0.0001	<0.0001	<0.0001	<0.0001	<0.0001	0.0039	0.0031	0.3025	0.0112	0.0143	<0.0001	0.9998	0.0003	0.0002
*L delbrueckii* ATCC 11842	0.0115	<0.0001	<0.0001	<0.0001	<0.0001	0.0008	<0.0001	<0.0001	<0.0001	<0.0001	<0.0001	<0.0001	0.9974	<0.0001	<0.0001	<0.0001	0.003	<0.0001	<0.0001	0.0002	0.5484	0.0768	0.0011	0.0085	0.6132

**Table 4:** Statistic analysis of pathobiont.

Bacterial strain	Media vs. Formula	Media vs. Fresh Milk	Media vs. Donor Milk	Media vs. Fresh + Fortifier	Media vs. Donor + Fortifier	Formula vs. Fresh Milk	Formula vs. Donor Milk	Formula vs. Fresh + Fortifier	Formula vs. Donor + Fortifier	Fresh Milk vs. Donor Milk	Fresh Milk vs. Fresh + Fortifier	Fresh Milk vs. Donor + Fortifier	Donor Milk vs. Fresh + Fortifier	Donor Milk vs. Donor + Fortifier	Fresh + Fortifier vs. Donor + Fortifier	Media vs. Iron	Media vs. Thickner	Media vs. Vitamins	Media vs. NaCl	Iron vs. Thickner	Iron vs. Vitamins	Iron vs. NaCl	Thickner vs. Vitamins	Thickner vs. NaCl	Vitamins vs. NaCl
*S. epidermidis* ATCC 51025	<0.0001	<0.0001	0.0006	<0.0001	<0.0001	0.0518	0.0056	<0.0001	<0.0001	0.7665	<0.0001	<0.0001	<0.0001	<0.0001	0.1195	0.0006	0.0001	>0.9999	0.1351	0.7902	0.0005	0.0236	0.0001	0.0044	0.1103
*S. agalactiae* ATCC 13813	0.0468	0.9281	0.7973	<0.0001	<0.0001	0.211	0.3333	0.0025	<0.0001	0.9994	<0.0001	<0.0001	0.0001	<0.0001	0.1356	0.0488	0.0287	0.034	0.0085	0.9965	0.9992	0.7834	>0.9999	0.9259	0.8879
*P. aeruginosa* CB1	<0.0001	0.4804	<0.0001	<0.0001	<0.0001	<0.0001	0.1035	0.7653	0.0338	<0.0001	<0.0001	<0.0001	0.0112	0.0003	0.279	0.2194	0.9997	0.9974	0.1401	0.2805	0.1387	0.0042	0.9859	0.1071	0.2215
*P. vulgaris* CB1	<0.0001	<0.0001	<0.0001	<0.0001	<0.0001	<0.0001	0.0036	0.9687	0.1722	0.2186	<0.0001	<0.0001	0.0011	<0.0001	0.4837	0.0001	<0.0001	<0.0001	0.0036	0.0003	0.0004	0.1075	0.9996	<0.0001	<0.0001
*E. cloacae* ATCC 2701	<0.0001	0.005	0.0013	<0.0001	<0.0001	0.011	0.0447	0.0003	<0.0001	0.9556	<0.0001	<0.0001	<0.0001	<0.0001	0.9403	0.0057	0.0003	0.0008	0.8143	0.2711	0.617	0.0013	0.9493	<0.0001	0.0002
*E. coli* K12	<0.0001	0.0103	<0.0001	<0.0001	<0.0001	<0.0001	0.5573	0.2327	0.9534	<0.0001	<0.0001	<0.0001	0.9792	0.9501	0.642	0.0004	0.9814	0.0093	<0.0001	0.0002	<0.0001	<0.0001	0.0209	<0.0001	<0.0001
*A. baumannii* ATCC 747	<0.0001	<0.0001	<0.0001	<0.0001	<0.0001	<0.0001	<0.0001	<0.0001	<0.0001	0.9992	<0.0001	<0.0001	<0.0001	<0.0001	0.9819	0.0065	<0.0001	0.0247	<0.0001	0.0009	0.8979	<0.0001	0.0003	<0.0001	<0.0001
*E. faecalis* ATCC 29212	<0.0001	<0.0001	<0.0001	<0.0001	<0.0001	<0.0001	<0.0001	0.144	0.3307	0.204	0.0016	<0.0001	<0.0001	<0.0001	0.0041	<0.0001	<0.0001	<0.0001	0.003	0.4725	0.0935	<0.0001	0.0069	<0.0001	0.0013
*K. pneumoniae* CB1	<0.0001	<0.0001	<0.0001	<0.0001	<0.0001	0.8259	<0.0001	<0.0001	<0.0001	<0.0001	<0.0001	<0.0001	<0.0001	<0.0001	0.5128	0.0002	<0.0001	<0.0001	0.8841	<0.0001	0.006	0.0005	<0.0001	<0.0001	<0.0001
*K. aerogenes* NCMB 10102	<0.0001	0.0367	0.0001	<0.0001	<0.0001	<0.0001	0.0043	<0.0001	<0.0001	0.0317	<0.0001	<0.0001	<0.0001	<0.0001	0.0179	0.0055	0.6872	0.099	0.9859	0.0406	0.3779	0.0115	0.5679	0.9179	0.2061

## Data Availability

The data that support the findings of this study are available on request from the corresponding author.
